# Testcross performance and combining ability of intermediate maturing drought tolerant maize inbred lines in Sub-Saharan Africa

**DOI:** 10.3389/fpls.2024.1471041

**Published:** 2024-11-28

**Authors:** Kulai Amadu Manigben, Yoseph Beyene, Vijay Chaikam, Pangirayi B. Tongoona, Eric Y. Danquah, Beatrice E. Ifie, Isaiah Aleri, Andrew Chavangi, Boddupalli M. Prasanna, Manje Gowda

**Affiliations:** ^1^ International Maize and Wheat Improvement Center (CIMMYT), World Agroforestry Centre (ICRAF), United Nations Avenue, Nairobi, Kenya; ^2^ West Africa Centre for Crop Improvement (WACCI), University of Ghana, Accra, Ghana; ^3^ Maize Improvement Program (MIP), The Council for Scientific and Industrial Research (CSIR)-Savanna Agricultural Research Institute, Tamale, Ghana

**Keywords:** drought, grain yield, general combining ability, line-by-tester design, specific combining ability, maize

## Abstract

Drought is a major constraint on maize (*Zea mays* L.) production and productivity in Sub-Saharan Africa (SSA). The increase in frequency and severity of drought, driven by climate change, is expected to worsen in the future. These occurrences are likely to adversely affect maize production and productivity, threatening the economic and social stability of millions of smallholder farmers. Understanding the genetics of hybrid performance under drought stress is crucial for designing breeding strategies to develop high-yielding hybrids. This study aimed to (i) evaluate the performance of three-way cross hybrids developed from elite inbred lines, including several drought-tolerant lines, using a line-by-tester mating design, and (ii) estimate the general combining ability (GCA) and specific combining ability (SCA) effects of the tropical maize inbred lines under managed drought and optimum conditions. A total of 265 maize inbred lines from the CIMMYT global maize breeding program were used as parents and crossed to six single cross testers to generate 795 testcross hybrids. These hybrids, along with six commercial hybrids as a check, were evaluated under managed drought and optimum conditions. Significant (*p* < 0.001) variations were observed among genotypes and genotypes-by-environment interactions (GEIs) for grain yield and other traits. There was a preponderance of GCA variance (lines and tester) over SCA variance, indicating that additive effects were more important in determining grain yield and other key traits under both managed drought and optimum conditions. Ten inbred lines (S2_8, S10_1, S6_4, S10_14, S2_14, S10_15, S8_7, S2_3, S8_15, and S13_5) with desirable GCA effects for grain yield and other traits were identified. Fourteen testcross hybrids were identified with high grain yield and desirable agronomic traits under both drought and optimum conditions. The identified lines and hybrids are useful sources to be used in breeding and deploying as stress-tolerant hybrids. High correlations observed between observed and GCA-predicted hybrid performance suggest the possibility to evaluate more hybrids with fixed resources. The study demonstrates that it is feasible to obtain high-yielding and drought-tolerant lines and hybrids. These testcross hybrids should undergo rigorous on-farm trials to ensure consistent performance before commercialization and release. Deploying these hybrids could help in mitigating the effects of drought stress in SSA and contribute to improved maize productivity in the region.

## Introduction

Maize (*Zea mays* L.) is the most widely consumed cereal crop and is grown on more than 40 million hectares of arable land in Sub-Saharan Africa (SSA) (Cairns et al., 2021). Maize contributes more than 30% of the daily calorie intake (varying from 50 to 30 g/person/day) (Prasanna et al., 2020b; Erenstein et al., 2022). Millions of households in SSA are dependent on maize for food and nutritional security and for providing income for securing their basic necessities of life (Ekpa et al., 2018; Santpoort, 2020; Poole et al., 2021; Food and Agriculture Organization (FAO), 2022).

Currently, maize yields in SSA are extremely low and exhibit significant variability, with an average yield ranging between 1 and 3 t/ha (Prasanna et al., 2022), which is significantly lower than the global average of 5 t/ha. The variability in maize yields in the maize-producing regions of SSA is attributed to a combination of biotic and abiotic stresses (Cairns and Prasanna, 2018; Prasanna et al., 2021; Ndlovu et al., 2022). With frequent occurring drought in the region being a key limiting abiotic factor, its negative impact makes it quite challenging to fully unlock the maximum yield potential of the maize (Pinho et al., 2022). Drought is expected to increase in both frequency and severity, which further exacerbates the declining yields in maize production zones in SSA, due to the continued pressure of climate change, rising temperatures, and irregular rainfall distribution (Cairns et al., 2021; Nurmberg et al., 2022; Cooper and Messina, 2023). Approximately 40% of the “maize belt” in SSA encounters periodic drought, leading to significant yield losses ranging between 10% and 25%. An additional 25% of the area is severely impacted by recurrent drought, which directly contributes to substantial harvest yield losses of up to 50% (Fisher et al., 2015). Recent studies have shown that drought stress is expected to inflict maize yield losses of up to 1.7% for each degree day spent above 30°C (Zhao et al., 2017; Ray et al., 2019; Prasanna et al., 2021).

The magnitude of drought-induced yield loss in maize varies from 30% to 90%, which largely depends on the maize growth stage, type of variety, and the duration/intensity of water deficit (Mcmillen et al., 2022; Prasanna et al., 2020a; Sheoran et al., 2022). It is therefore not surprising that smallholder farmers in SSA have high preference and demand for drought-tolerant maize varieties for cultivation. Farmers and other actors in the maize value chain consider drought tolerance as a “must-have” trait and has become a key breeding objective for most maize breeding program in SSA (Beyene et al., 2021).

The International Maize and Wheat Improvement Center (CIMMYT) in collaboration with national agriculture research organizations developed and released a wide range of high-yielding drought-tolerant maize inbred lines, hybrids, and open pollinated varieties for different maturity groups and adaptations in SSA. These contributed to significant genetic gain for grain yield (GY) under managed drought (32.5 kg ha^−1^ year^−1^) and random drought stress (22.7 kg ha^−1^ year^−1^) conditions (Badu-Apraku and Fakorede, 2017; Masuka et al., 2017; Prasanna et al., 2020a; Prasanna et al., 2022). Although significant gains in breeding for drought-tolerant varieties have been achieved, these gains are inadequate to meet future demand due to population growth, global climate change with unpredictable rainfall pattern, and rising temperature, particularly in SSA (Masuka et al., 2017; Cairns and Prasanna, 2018; Barbosa et al., 2021). Therefore, genetic improvement and breeding for improved tolerance in tropical maize germplasm are highly relevant strategies to continue to guarantee food security, reduce smallholder farmers’ vulnerability to drought, and mitigate against climate change.

Development of drought-tolerant maize varieties largely depends on the availability of genetic variation and carefully selecting appropriate parental lines that show high performance *per se* and can transmit favorable genes/alleles to their progeny. For the effective and judicious use of elite maize germplasm, basic genetic information about the breeding value of a new set of inbred lines can help to accelerate the development of high-yielding cultivars with desirable traits (e.g., drought tolerance). This is critical in establishing an appropriate breeding plan or strategy that can be employed to effectively improve the development of productive hybrids with improved drought tolerance.

Combining ability is the ability of parental lines to effectively pass on genes/alleles to their progenies. Genetic mating designs including diallel mating designs (Griffing, 1956); North Carolina design I, II, and III (Comstock and Robinson, 1948); and line-by-tester mating design (Hallauer et al., 2010) are commonly used for the evaluation of the combining ability of new inbred lines and for providing key information about the genetic control of desirable traits. Among these mating designs, the line-by-tester design involves crossing few known testers to a large number of inbred lines that allow one to estimate the lines’ combining ability and breeding value (Acquaah, 2012). Early-stage testcross evaluation using the line-by-tester design enables the identification of inbred lines that are good combiners with high or low *per se* performance, which save time and money by helping breeders to discard poor combiners at the early stage of testing in breeding.

Over the years, numerous studies have been conducted to evaluate maize inbred lines for their combining ability, breeding values, *per se* performance, and gene activity under drought and optimum conditions (Betrán et al., 2003b; Makumbi et al., 2011; Oyekunle, 2014; Badu-Apraku et al., 2015; Trachsel et al., 2016; Beyene et al., 2017; Osuman et al., 2022; Sang et al., 2022; Matongera et al., 2023). Using the line-by-tester design, Oluwaseun et al. (2022) and Ertiro et al. (2017) reported that additive gene activity was more important in regulating GY and other traits under drought stress. In contrast, Annor et al. (2019), using the line-by-tester design, reported that non-additive gene effects were more important than additive gene action in controlling GY under drought stress. The conflicting results on gene activity controlling yield and other traits under drought conditions can be attributed to variations in the genetic background of inbred lines and the environment conditions in which the lines are evaluated. Therefore, it is crucial for breeders to determine the combining ability of a new set of maize inbred lines and their gene action derived from populations of diverse sources. This enables the selection of superior inbred lines that contribute desirable alleles/genes to their crosses, which can be used as donors in genetic improvement for drought tolerance and the production of highly productive hybrids. In eastern and southern Africa, CIMMYT is developing improved drought-tolerant and other multiple stress-resilient, high-yielding potential inbred lines, which helps to achieve high genetic gain under smallholder farming conditions. Understanding the genetic potential of newly developed inbred lines in hybrid combinations is critical for their effective utilization. Therefore, combining ability analysis of the newly developed elite drought-tolerant inbred lines is a vital tool to identify and select the most desirable inbred lines for the development of high-yielding and disease-resistant hybrids adapted to the target environments in the region. The objectives of this study were to (i) evaluate the performance of three-way cross hybrids under drought and optimum conditions and (ii) estimate the GCA and SCA effects of a new set of tropical maize inbred lines for GY and other agronomic traits under managed drought and optimum conditions.

## Materials and methods

### Plant materials and hybridization

A total of 265 new inbred lines derived from CIMMYT’s east Africa mid-altitude medium maturity breeding program (EA-PP1) were used in the present study. The selected 265 lines were developed from a set of elite drought donor lines in an elite-by-elite line combination. Specific drought donors like LapostaSequia-C7-F71, LapostaSequia-C7-F78, LapostaSequia-C7-F103, LapostaSequia-C7-F180, CML386, and DTPWC9 were used to develop these lines. In addition to drought tolerance, lines with good GCA and *per se* performance for high yield potential as well as resistance to several foliar diseases were also included in this study. An incomplete line-by-tester design (Kempthorne, 1957) was used to generate 795 testcrosses by crossing 265 lines to six single-cross testers. A total of 93 lines belong to CIMMYT heterotic group A while the remaining 172 lines belong to heterotic group B. All lines were crossed to three testers from opposite heterotic groups (**Supplementary Table S1**). Pollen from each line was used to manually pollinate the single cross testers. The testcrosses were formed during the short rainy season (October–March) in the Kiboko maize research station in Kenya. The inbred lines were selected through rigorous phenotypic evaluations in the breeding nurseries and screened for foliar diseases. The testers used in the study are good combiners and are used as a female parent to adapt the three-way hybrid to mid-altitude environments. Detailed information on the pedigree of the inbred line is presented in **Supplementary Table S1**.

### Experimental design and trial management

A total of 795 testcrosses were evaluated in a multilocation trial under optimum and managed drought conditions. The experiments were interconnected by six commercial hybrid checks (DK8031, H513, PH3253, Pioneer 3253, DH04, and WH505). An Alpha (0,1) lattice experimental design with two replications was used. Each genotype was planted in two-row plots of 5 m in length, spaced at 0.75 m between rows and 0.25 m between hills. Each hill was planted with two seeds and later thinned to one plant per hill at 3 weeks after seedling emergence to adjust the final plant population to 53,333 plants/ha. Basal fertilizer application was performed at planting using di-ammonium phosphate (DAP) fertilizer at the rate of 60 kg N and 60 kg P_2_O_5_ per hectare. Six weeks after emergence, all experiments were top dressed with nitrogen fertilizer at the rate of 60 kg N/ha. All the experiments were kept weed-free by manual weeding and herbicide control.

The testcrosses were evaluated in Kenya at four locations under optimum management (Kakamega, Kiboko, Kirinyaga, and Shikutza) and at three locations (Kiboko, Homabay, and Mtwapa) under drought stress conditions (**Table 1**). Drought stress experiments were conducted in the off season where it does not rain during cropping period. The drought stress fields are irrigated with a drip irrigation system. Irrigation was applied twice weekly for 3 to 5 h depending on the potential evapotranspiration (Trachsel et al., 2016). Irrigation of the trials was stopped at ~750 GDD after planting (2 weeks before the anticipated date of flowering) to induce drought stress (Trachsel et al., 2016; Ertiro et al., 2017). The well-watered or optimum trials were conducted in the main season under rainfed conditions. The trials were also supplemented with irrigation throughout the growth cycle to avoid drought stress. The management of drought stress experiment and phenotyping were according to the procedures outlined in the drought phenotyping protocols by CIMMYT (Zaman-Allah et al., 2016; Zaidi, 2019).

**Table 1:** Description of trial location and management condition.

Location	Coordinates	Elevation (masl)	Management	Rainfall (mm/year)	Mean temp (°C)
Kiboko	2.2103° S, 37.7231° E	925	Optimum and drought	530	24.7
Homabay	0.5350° S, 34.4531° E	1,131	Drought	1,500	21.8
Mtwapa	3.9386° S, 39.7498° E	25	Drought	997	26
Kirinyaga	0.6591° S, 37.3827° E	1,550	Optimum	1,688	19.2
Kakamega	0.28° N, 34.75° E	1,535	Optimum	1,742	20.8
Shikutsa	0.28° N, 34.75° E	1,560	Optimum	1,700	21.62

### Data collection

Data were collected per plot for each experiment under managed drought and optimum conditions for days to 50% anthesis (AD, as number of days after planting when 50% of the plants per plot shed pollen), days to 50% silking (SD, as number of days after planting when 50% of the plants per plot show silks), anthesis–silking interval (ASI) (as the difference between AD and SD), plant height (PH, measured as the length in centimeters from the base of a plant to the insertion of the first tassel branch of the same plant for 10 representative plants per plot), and ear height (EH, measured as the length in centimeters from the base of a plant to the internode of the top ear of the same plant for 10 representative plants per plot). At harvest, field weight [weight of dehusked ears (cobs) in kilograms per plot] and grain moisture (MOI, measured using a moisture meter on grain sampled from the center of five representative ears per plot) were recorded. GY was calculated using the field weight of ears per plot and a shelling percentage of 80, and adjusted to a moisture content of 12.5%. For drought trials, all trait measurements were performed according to the procedures outlined in the drought phenotyping protocols by CIMMYT (Zaman-Allah et al., 2016; Zaidi, 2019).

### Phenotypic data analysis

The restricted maximum likelihood method was utilized to conduct analysis of variance for each trial as well as a combined analysis across environment years using the META-R statistical package (Alvarado et al., 2020). The following linear mixed model was used:


$${\text{Y}}_{\text{ijkb}}=\;\text{μ}+\;{\text{E}}_{\text{j}}+\;\text{R}{\left(\text{E}\right)}_{\text{kj}}+\text{B}{\left(\text{RE}\right)}_{\text{bkj}}+{\text{G}}_{i}+{\text{GE}}_{\text{ij}}+{\text{ϵ}}_{\text{ijkb}}$$

where $Y_{ijkb}$ is the observed trait of interest; μ is the overall mean; $E_{j}$ is the effect of $j^{th}$ environment; $R{\left(E\right)}_{kj}$ is the effect of $k^{th}$ replication within $j^{th}$ environment; $B{\left(RE\right)}_{bkj}$ is the effect of $b^{th}$ block within environment; $R{\left(E\right)}_{kj}$ is the effect of $k^{th}$ replication within $j^{th}$ environment; $B{\left(RE\right)}_{bkj}$ is the effect of $b^{th}$ block within $k^{th}$ replication in $j^{th}$ environment; ${\text{G}}_{i}$ is the effect of the $i^{th}$ genotype; ${\text{GE}}_{ij}$ is the $i^{th}$ genotype by $j^{th}$ environment interaction and $\epsilon_{ijkb}$ is effect of the residual error. Residuals are assumed as independent and identically distributed, $\epsilon ∼\;iidN(0,\epsilon_{ijkb}^{2}$ ). Heritability in the broad sense (Hallauer et al., 2010) was computed as:


H2=σg2  σg2+σge2e+σϵ2re      

where $\sigma_{g}^{2}$ , $\sigma_{ge}^{2}$ and $\sigma_{\epsilon }^{2}$ is the genotype variance, genotype-by-environment interaction variance, and residual variance, respectively. The number of replications and environments is denoted by *r* and *e*, respectively. The correlation between traits under managed drought and optimum conditions was calculated using the “*cor*” function in R (R Core Team, 2023). A correlation heatmap and the distribution of phenotypic values of the traits were generated using the *ggplot2* R package.

### Line-by-tester mating design analysis

Line-by-tester analysis was conducted by excluding the checks from the analysis. The total variance of the testcrosses values was partitioned into the GCA variance due to the line and tester as well as the specific combining ability variance (SCA) due to line × tester (Trachsel et al., 2016; Liu et al., 2021). Data were analyzed using the AGD-R version 4 software (Rodríguez et al., 2015). Analyses were performed for within (Equation 1) and across locations (Equation 2) using the joint linear mixed model as:


(1) $${\text{Y}}_{\text{ijkm}}=\text{μ}+{\text{R}}_{\text{k}}+\text{B}{\left(\text{R}\right)}_{\text{km}}+{\text{L}}_{i}+{\text{T}}_{\text{j}}+\text{L}{\text{T}}_{i\text{j}}+{\text{ϵ}}_{i\text{jkm}}$$

where $Y_{ijkm}$ is the observed value, μ is the overall mean; $R_{k}$ is the effect of $k^{th}$ replication; $B{\left(R\right)}_{km}$ is the effect of $m^{th}$ incomplete block within $k^{th}$ replication; ${\text{L}}_{i}$ is the GCA effect of the $i^{th}$ line; ${\text{T}}_{j}$ is the GCA effect of the $j^{th}$ tester; $LT_{ij}$ is the SCA effect of the $i^{th}$ line by $j^{th}$ tester, and $\epsilon_{ijkm}$ is the residual effect assumed to be independent and identically distributed as $\epsilon ∼\;iidN(0,\epsilon_{ijkm}^{2}$ ).


(2) $$\begin{array}{c} {\text{Y}}_{i\text{jdkm}}=\;\text{μ}+\;{\text{E}}_{\text{d}}+\;\text{R}{\left(\text{E}\right)}_{\text{kd}}+\text{B}{\left(\text{RE}\right)}_{\text{mkd}}+{\text{L}}_{i}+{\text{T}}_{\text{j}}+\text{L}{\text{T}}_{i\text{j}}\\ \;\;\;+\text{L}{\text{E}}_{i\text{d}}+\text{T}{\text{E}}_{\text{jd}}+\text{LT}{\text{E}}_{i\text{jd}}+{\text{ϵ}}_{i\text{jdkm}} \end{array}$$

where $Y_{ijdkm}$ is the response value, μ is the overall mean; $E_{d}$ is the effect of $d^{th}$ environment; $R{\left(E\right)}_{kd}$ is the effect of $k^{th}$ replication within $d^{th}$ environment; $\;B{\left(RE\right)}_{mkd}$ is the effect of $m^{th}$ incomplete block within $k^{th}$ replication in $d^{th}$ environment; ${\text{L}}_{i}$ is the GCA effect of the $i^{th}$ line; ${\text{T}}_{j}$ is the GCA effect of the $j^{th}$ tester; $LT_{ij}$ is the SCA effect of the $i^{th}$ line by $j^{th}$ tester; $LE_{id}$ is the GCA effect of $i^{th}$ line interacting with $d^{th}$ environment; $TE_{jd}$ is the GCA effect of $j^{th}$ tester interacting with $d^{th}$ environment; $LTE_{ijd}$ is the SCA effect of $i^{th}$ line by $j^{th}$ tester interacting with $d^{th}$ environment and $\epsilon_{ijdkm}$ is the residual effect assumed to be independent and identically distributed, $\epsilon ∼\;iidN(0,\epsilon_{ijdkm}^{2}$ ).

The effects of environment and replication within the environment were considered fixed, while line, tester, line × tester, and their corresponding interaction with environment and block within environment within replication were considered random. The restricted maximum likelihood procedure was used to estimate all variance components (Rodríguez et al., 2015). Tests of the significance of the variance component estimates and model comparison were performed using the likelihood ratio test (Stram and Lee, 1994; Giampaoli and Singer, 2009).

### Estimation of GCA and SCA effects

The estimates of the GCA effects for line, tester, and SCA effects of the line-by-tester interaction were estimated according to the procedure outlined by Singh and Chaudhary (1985). To test the significance of GCA and SCA at the 5% level of significance, the values of GCA for each line and tester and the SCA values of each hybrid were divided by their corresponding standard error, to compute their respective *t*-values. The calculated *t*-values were then compared with the tabular *t*-values at their corresponding error degree of freedom (Singh and Chaudhary, 1985). To assess the relative significance of GCA and SCA effects, Baker’s ratio was estimated using the variance of GCA of lines and testers and SCA based on the following formula:


$$Baker^{\prime }s\;ratio=\frac{2\sigma^{2}GCA}{2\sigma^{2}GCA+\sigma^{2}SCA}$$

where $\sigma^{2}GCA$ is the GCA variance of lines or tester and $\sigma^{2}SCA$ is the SCA line × tester variance. The closer the ratio to unity, the greater the predictability of a hybrid performance based on GCA effects alone (Baker, 1978).

The proportional contribution of total genetic variance due to lines, tester, and the interaction between lines and tester was computed as suggested by Singh and Chaudhary (1985). Pearson’s correlation of testcross hybrid performance (TCP) with the sum of GCA effects of both the parents r(GCA, TCP) using a leave-one-hybrid-out cross-validation was explained in detail by Schrag et al. (2009). All analyses were performed using the ASReml-R software version 3.0 (Butler et al., 2009).

## Results

### Analysis of variance under drought and optimum conditions

**Figure 1:**
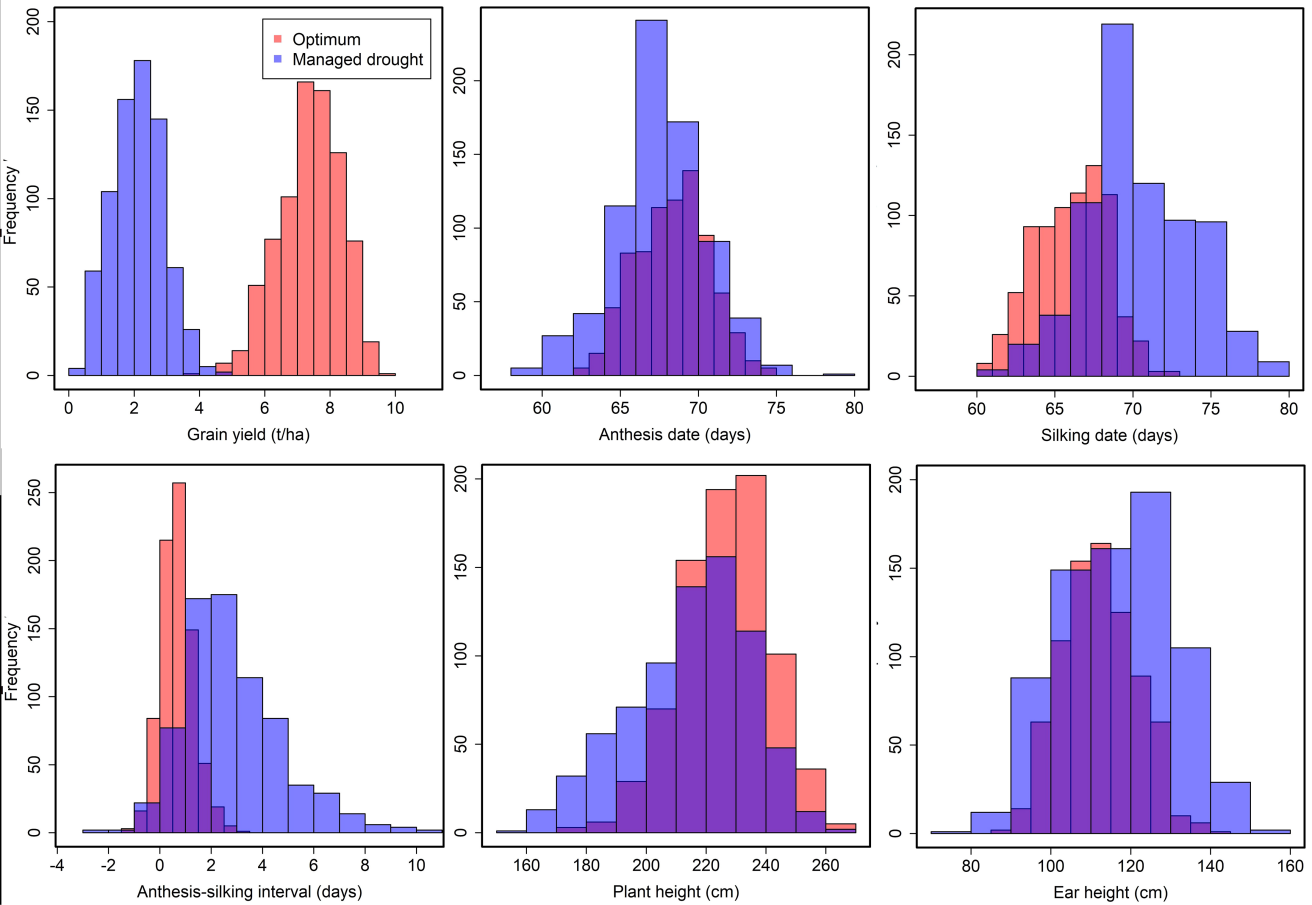
Frequency distribution of grain yield, anthesis date, silking date, anthesis–silking interval, plant height, and ear height recorded under managed drought and optimum conditions. Blue color denotes traits measured under drought stress and orange color denotes trait performance under optimum condition.

Individual location analyses for GY under managed drought and optimum conditions revealed significant (*p* < 0.05) genotypic variance and the heritability estimates ranged from 0.17 to 0.73 (**Supplementary Table S2**). Results of combined analysis across locations under optimum and managed drought conditions are shown in **Table 2**. The analysis showed highly significant (*p* < 0.01) genotypic variance among the testcrosses for all the traits indicating that genetic variation among testcross hybrids was abundant. The ratio of genotype to genotype-by-environment interaction (GEI) variance for GY was higher in the optimum condition compared to managed drought stress condition, suggesting that GEI interaction was greater under drought conditions. The GEI revealed a highly significant (*p* < 0.01) variance for GY, AD, SD, ASI, PH, and EH under optimum and drought conditions. The coefficients of variation for all the traits under optimum and drought conditions were below 25% except for ASI. The estimates of broad-sense heritability for all traits were relatively higher under optimum conditions (0.53 to 0.86) than under drought stress conditions (0.25 to 0.85). The low experimental coefficient of variation and high broad-sense heritability for most of the traits reflected good experimental accuracy. A wide range of phenotypic variation was observed among the testcrosses for all the traits under drought and optimum conditions (**Figure 1**). Drought stress considerably reduced GY, AD, and SD compared to optimum. Furthermore, drought stress prolonged the interval between AD and SD in the testcrosses. Plant height was reduced by drought stress compared to the optimum condition.

**Table 2:** Variance component, coefficient of variation, heritability of grain yield, and other traits under optimum and drought conditions.

Source of variance	GY	AD	SD	ASI	PH	EH
	Managed drought					
Genotype Variance	0.06***	4.71***	4.98***	0.49*	140.23***	86.29***
Gen × Env Variance	0.28***	0.97***	1.68***	0.69***	56.48***	28.48***
Residual Variance	0.50	2.90	5.40	2.20	260.30	103.40
CV (%)	22.2	2.7	3.6	95.9	8.6	11
Heritability	0.25	0.85	0.77	0.46	0.69	0.76
	Optimum condition					
Genotype Variance	0.31***	4.35***	3.69***	0.21***	127.61***	53.21***
Gen × Env Variance	0.58***	1.06***	1.38***	0.19***	133.33***	74.36***
Residual Variance	2.6	7.5	9.5	1.5	316.6	121.5
CV (%)	22.3	4	4.5	149	7.5	9.4
Heritability	0.53	0.86	0.81	0.60	0.75	0.73

* and *** Significant at *p* < 0.05, < 0.01, and < 0.001 probability levels, respectively. ɈGY, grain yield; AD, days to 50% anthesis; SD, days to 50% silking; ASI, anthesis–silking interval; H, plant height; EH, ear height.

### Performance of testcrosses

The mean performance of GY and other agronomic traits for the 20 selected best testcross hybrids and six commercial checks under managed drought and optimum conditions is presented in **Tables 3** and **4**, respectively. Under drought stress, GY averaged 2.15 t/ha, with a range of 0.41 to 4.89 t/ha. In contrast, under optimum conditions, the average GY was significantly higher at 7.39 t/ha, ranging from 3.95 to 9.77 t/ha. Under drought stress, it took 58 to 79 days (average, 67 days) to reach 50% anthesis (AD). Under optimum conditions, AD averaged 68 days, ranging from 62 to 74 days. For silking (SD), it took 60 to 79 days (average, 70 days) under drought stress and 60 to 72 days (average, 66 days) under optimum conditions. The ASI averaged 2.8 days (range, −2 to 10 days) under drought stress and 1 day (range, −1.4 to 3.1 days) under optimum conditions (**Tables 3**, **4**). However, under optimum conditions (**Table 3**), PH varied from 172 to 265 cm with a mean of 226.4 cm under optimum conditions. Under managed drought conditions, it ranged from 153.2 to 263.1 cm with an average of 214.7 cm. Generally, plant and ear height were taller under optimum conditions than under drought stress conditions (**Tables 3**, **4**).

**Table 3:** Mean performance of grain yield and other agronomic traits in 30 testcross hybrids (20 best and 10 worst) plus six checks tested under drought.

Testcross	GY	AD	SD	ASI	PH	EH
S13_5 × T2	4.89	64.89	65.40	0.47	217.84	110.06
S13_5 × T5	4.58	65.42	65.34	−0.05	218.53	101.19
S7_9 × T2	4.50	66.44	67.00	0.57	243.67	126.01
S13_3 × T2	4.39	64.72	67.92	3.01	221.20	105.12
S13_3 × T3	4.30	64.58	67.85	3.24	216.72	97.82
S13_6 × T3	4.26	65.28	65.16	−0.17	196.21	99.35
S13_5 × T3	4.12	65.78	65.75	−0.03	211.64	106.33
S13_7 × T3	4.00	64.73	65.09	0.12	217.46	107.58
S13_13 × T5	3.89	67.84	69.13	1.12	227.00	118.59
S13_14 × T5	3.89	65.81	65.85	0.01	211.92	95.15
S13_3 × T5	3.88	65.95	69.02	3.07	214.93	100.71
S7_2 × T5	3.86	67.82	68.47	0.59	240.83	131.54
S13_12 × T3	3.86	65.82	66.47	0.59	215.33	106.54
S13_6 × T5	3.83	65.17	65.62	0.46	214.09	108.80
S1_15 × T1	3.79	64.98	66.43	1.49	243.09	106.39
S6_11 × T4	3.75	66.56	69.22	2.51	210.16	111.43
S13_4 × T3	3.74	65.25	68.05	2.74	214.26	106.01
S13_1 × T5	3.73	64.45	66.45	1.97	217.68	101.52
S13_13 × T2	3.72	68.28	69.78	1.53	212.49	118.59
S13_12 × T5	3.72	67.69	68.68	1.08	201.87	95.84
S16_22 × T5	0.62	73.01	78.83	6.87	170.78	107.41
S12_1 × T2	0.61	68.18	73.73	5.58	176.17	102.37
S11_7 × T5	0.60	64.98	NA	NA	187.21	109.53
S12_9 × T2	0.56	67.84	75.98	8.07	184.14	116.70
S12_12 × T5	0.56	72.41	74.92	2.55	202.17	127.91
S11_7 × T3	0.54	62.04	74.09	8.06	164.11	94.69
S16_5 × T3	0.53	72.09	79.45	7.45	217.11	132.48
S12_12 × T3	0.51	72.78	74.78	2.03	185.49	110.59
S12_14 × T5	0.50	65.43	76.36	NA	197.76	113.92
S12_4 × T5	0.46	67.82	74.22	8.10	189.74	109.39
DH04	1.72	66.53	72.21	4.72	209.89	114.18
DK8031	1.30	68.00	74.25	5.39	208.87	114.50
H513	1.87	65.56	68.24	2.66	208.43	116.65
PH3253	1.92	67.80	71.83	4.01	231.75	123.87
Pioneer3253	1.33	69.53	74.37	4.08	207.10	122.40
WH505	2.62	68.40	71.99	3.53	221.88	117.70
Grand mean	2.15	67.56	70.36	2.78	214.71	116.88
Minimum	0.41	58.92	60.16	−2.04	153.21	75.98
Maximum	4.89	79.31	79.45	9.99	263.51	156.24
SED	0.93	1.55	1.93	0.82	11.36	8.06

GY, grain yield; AD, days to 50% anthesis; SD, days to 50% silking; ASI, anthesis–silking interval; H, plant height; EH, ear height; OPT, optimum condition; DS, drought condition.

**Table 4:** Mean performance of grain yield and other agronomic traits in 30 testcross hybrids (20 best and 10 worst) plus six checks tested under optimum conditions.

Testcross	GY	AD	SD	ASI	PH	EH
S2_8 × T1	9.77	67.60	65.61	1.00	246.88	120.47
S4_10 × T1	9.46	69.74	67.92	1.13	243.50	118.12
S6_4 × T1	9.39	68.29	66.75	0.76	234.93	114.57
S6_4 × T4	9.35	68.01	67.60	1.26	245.49	122.63
S2_8 × T6	9.34	67.80	65.04	0.25	241.65	116.97
S2_14 × T4	9.33	66.16	64.28	1.00	232.86	104.27
S10_1 × T5	9.22	68.57	65.70	0.12	250.14	127.61
S8_7 × T3	9.19	66.05	63.44	0.58	245.90	118.81
S2_3 × T4	9.17	67.71	65.17	0.38	234.78	103.48
S2_9 × T1	9.12	66.32	63.85	0.51	238.12	112.54
S2_1 × T1	9.12	67.04	65.21	1.24	247.49	116.31
S8_15 × T5	9.12	64.96	63.02	1.41	248.46	112.93
S4_5 × T4	9.10	70.19	67.65	0.38	226.98	115.02
S4_10 × T4	9.09	70.13	68.25	1.00	233.63	113.40
S8_15 × T3	9.08	65.23	62.64	0.75	251.19	113.43
S13_8 × T3	9.07	70.65	67.38	−0.26	240.45	121.12
S6_11 × T4	9.05	65.52	63.32	0.01	227.88	113.07
S10_1 × T2	9.05	67.88	64.94	0.01	239.50	123.63
S8_11 × T5	9.03	67.08	64.23	0.25	249.12	121.20
S2_8 × T4	9.01	67.23	64.54	0.25	241.87	120.45
S9_7 × T3	5.16	68.62	68.82	2.47	212.63	106.82
S9_18 × T3	5.12	67.07	65.94	1.31	223.25	106.38
S9_11 × T3	5.12	68.12	66.32	1.52	211.15	99.40
S3_17 × T1	4.99	68.71	66.82	1.15	203.33	97.21
S3_13 × T1	4.99	71.53	69.18	0.55	209.48	103.19
S3_12 × T1	4.96	70.14	68.81	1.14	194.12	93.17
S3_8 × T4	4.94	69.13	66.81	1.13	193.47	97.98
S3_8 × T1	4.91	71.39	66.87	−0.70	202.55	104.00
S3_8 × T6	4.76	69.46	67.13	0.62	198.21	100.19
S9_11 × T2	4.52	68.44	67.93	1.85	218.43	101.89
DH04	5.29	67.96	66.25	1.41	225.16	111.45
DK8031	3.95	68.63	68.39	2.48	218.99	106.16
H513	6.44	67.93	65.46	0.97	234.40	122.84
PH3253	6.10	68.41	66.33	0.98	237.67	119.70
Pioneer3253	5.92	69.47	68.36	1.49	231.81	115.58
WH505	7.01	70.98	67.54	0.37	229.21	112.88
Grand mean	7.39	68.34	66.05	0.70	226.39	112.16
Minimum	3.95	62.13	60.32	−1.41	172.00	85.96
Maximum	9.77	74.67	72.30	3.11	265.00	141.64
SED	0.72	1.15	1.3	0.51	8.95	6.09

GY, grain yield; AD, days to 50% anthesis; SD, days to 50% silking; ASI, anthesis–silking interval; H, plant height; EH, ear height; OPT, optimum condition; DS, drought condition.

Under managed drought conditions, testcrosses S13_5 × T2 (4.9 t/ha), S13_5 × T5 (4.6 t/ha), S7_9 × T2 (4.50 t/ha), S13_3 × T2 (4.4 t/ha), and S13_3 × T3 (4.3 t/ha) are among the top-performing hybrids, with GY outperforming the best commercial check (WH505; **Table 3**). The yield performance of the best testcross hybrid under managed drought conditions was almost double that of the best commercial check (**Table 3**). Under optimum conditions, the best testcross hybrid (S2_8 × T1) outyielded the best commercial hybrid by 2.7 tons (**Table 4**). Overall, drought stress reduced the GY by 20%. Under the optimum condition (**Table 4**), testcross S2_8 × T1 (9.8 t/ha), S4_10 × T1 (9.46 t/ha), S6_4 × T1 (9.39 t/ha), S6_4 × T4 (9.35 t/ha), S2_8 × T6 (9.34 t/ha), and S2_14 × T4 (9.3 t/ha) are the best-performing hybrids. All the 20 best hybrids outyielded the best commercial check (WH505).

### Relationship between traits under drought and optimum conditions

**Figure 2:**
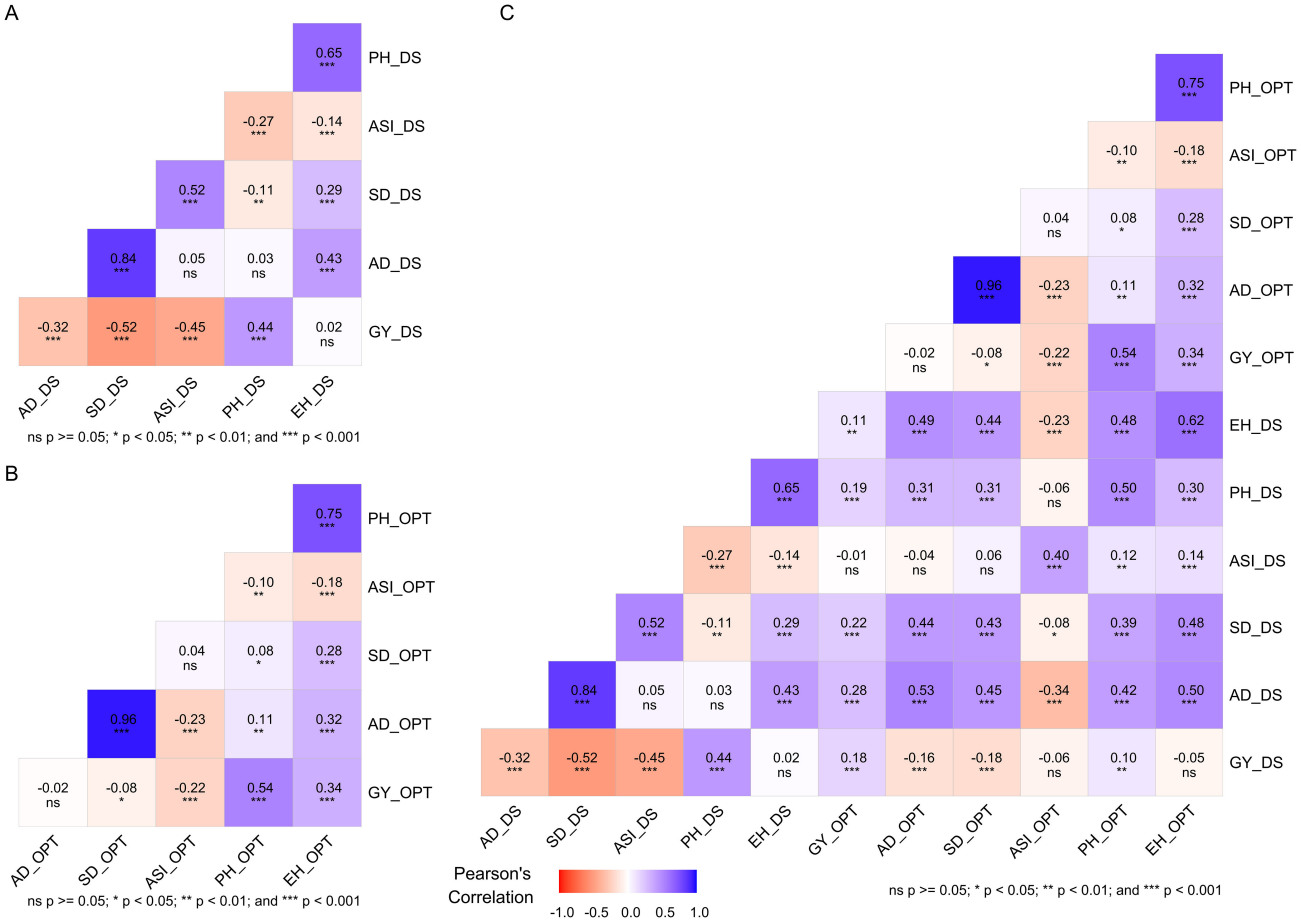
Pearson’s correlation between grain yield and other traits measured under drought stress **(A)**, under optimum condition **(B)**, and under both drought and optimum conditions **(C)**. The correlation level is color-coded according to the color key indicated on the scale. Correlations with *, **, and *** were significant at 0.05, 0.01, and 0.001 levels, respectively. AD, days to 50% anthesis; ASI, anthesis to silking interval; SD, silking date; GY, grain yield; PH, plant height; EH, ear height; DS, drought stress; OPT, optimum.

Insights into the relationships among traits is crucial for breeders when selecting suitable traits to incorporate in selection criteria. Significant (*p* < 0.001) coefficients of phenotypic correlation were observed among most traits across drought and optimum conditions (**Figures 2A–C**). Significant (*p* < 0.001) and positive correlations were identified between GY and PH and EH. PH and EH implied that selection improvement of these traits under optimum conditions could lead to an increase in GY (**Figure 2B**). Furthermore, significant (*p* < 0.001) and negative correlations observed between GY and AD, SD and ASI, and ASI with PH, EH, and AD under optimum conditions implied that that these traits are reliable selection indices for yield improvement in optimum conditions. A significant (*p* < 0.001) and negative correlation was observed between GY and AD_DS, SD_DS, and ASI_DS under drought stress. This implied that selection of GY alone selection may not be effective.

### Analyses of GCA and SCA variance

The genotypes and GEI variance components were significant at the 0.05% probability level for all traits under OPT and DS conditions (**Table 5**). The GCA variance for lines and testers were highly significant (*p* < 0.001) for majority of the traits under both DS and OPT conditions, indicating high genetic variation among these genotypes. In contrast, GCA variance for testers was not significant for GY under DS conditions. The SCA variance for line × tester interactions was not significant for all the traits under DS and OPT conditions (**Table 5**). Variances for line × environment and tester × environment interactions were significant (*p* < 0.001) for most of the traits under DS and OPT conditions. However, tester-by-environment interaction variances were not significant for GY, PH, and EH under DS conditions. SCA variances were not significant for most of the traits under DS and OPT conditions (**Table 5**). Baker’s ratio (**Tables 5**, **6**) was closer to or equal to 1 for all the traits under DS and OPT conditions.

**Table 5:** Partition of variance components for line by tester and Baker’s ratio of grain yield and other traits evaluated under drought and optimum conditions.

Variance components	GY	AD	SD	ASI	PH	EH
Drought stress						
$\sigma_{\text{G}}^{2}$	0.04*	4.92***	5.66***	0.53***	159.44***	92.08***
$\sigma_{\text{GC}{\text{A}}_{\text{L}}}^{2}$	0.36***	5.31***	6.18***	0.47***	187.18***	101.59***
$\sigma_{\text{GC}{\text{A}}_{\text{T}}}^{2}$	0.00	0.00***	0.06***	0.02***	3.22***	1.74***
$\sigma_{\text{SC}{\text{A}}_{\text{LT}}}^{2}$	0.00	0.00	0.09	0.00	0.00	0.00
$\sigma_{\text{G}\times \text{E}}^{2}$	0.32***	1.27***	1.72***	0.70***	49.49***	27.59***
$\sigma_{\text{GC}{\text{A}}_{\text{L}\times \text{E}}}^{2}$	0.02*	0.88***	1.32***	0.55***	60.10***	27.36***
$\sigma_{\text{GC}{\text{A}}_{\text{T}\times \text{E}}}^{2}$	0.00	0.17**	0.08*	0.06**	4.58	1.72
$\sigma_{\text{SC}{\text{A}}_{\text{LT}\times \text{E}}}^{2}$	0.00	0.13	0.00	0.00	0.00	0.00
$\sigma_{\text{ϵ}}^{2}$	1.01	1.99	3.51	1.54	187.48	76.65
**Baker’s ratio**	1.0	1.00	0.99	1.00	1.00	1.00
Optimum						
$\sigma_{\text{G}}^{2}$	0.27***	4.43***	3.75***	0.20***	128.33***	54.00***
$\sigma_{\text{GC}{\text{A}}_{\text{L}}}^{2}$	0.228***	4.417***	3.92***	0.203***	129.31***	49.14***
$\sigma_{\text{GC}{\text{A}}_{\text{T}}}^{2}$	0.015***	0.050***	0.009***	0.000***	4.36***	10.75***
$\sigma_{\text{SC}{\text{A}}_{\text{LT}}}^{2}$	0.01	0.00	0.00	0.00	1.07	0.58
$\sigma_{\text{G}\times \text{E}}^{2}$	0.56***	1.21***	1.56***	0.22***	143.23***	75.49***
$\sigma_{\text{GC}{\text{A}}_{\text{L}\times \text{E}}}^{2}$	1.031***	3.467***	3.93***	0.252***	208.37***	82.91***
$\sigma_{\text{GC}{\text{A}}_{\text{T}\times \text{E}}}^{2}$	0.046***	0.081***	0.23***	0.037***	4.32***	10.45***
$\sigma_{\text{SC}{\text{A}}_{\text{LT}\times \text{E}}}^{2}$	0.00	0.00	0.00	0.00	0.00	0.00
$\sigma_{\text{ϵ}}^{2}$	1.85	4.90	6.26	1.38	199.64	102.23
**Baker’s ratio**	0.99	1.00	1.00	0.99	1.00	1.00

*, **, and *** denote significance at *p* < 0.05, *p* < 0.01, and *P* < 0.001, respectively. GY, grain yield; AD, days to 50% anthesis; SD, days to 50% silking; ASI, anthesis–silking interval; H, plant height; EH, ear height. Ɉ $\sigma_{\text{G}}^{2}$ , genotype variance; $\sigma_{\text{GC}{\text{A}}_{\text{L}}}^{2}$ , GCA line variance; $\sigma_{\text{GC}{\text{A}}_{\text{T}}}^{2}$ , GCA Tester variance; $\sigma_{\text{SC}{\text{A}}_{\text{LT}}}^{2}$ , SCA Line × Tester variance; $\sigma_{\text{GC}{\text{A}}_{\text{L}\times \text{E}}}^{2}$ , GCA Line by Environment interaction variance; $\sigma_{\text{GC}{\text{A}}_{\text{T}\times \text{E}}}^{2}$ , GCA Tester by Environment interaction variance; $\sigma_{\text{SC}{\text{A}}_{\text{LT}\times \text{E}}}^{2}$ , SCA Line by Tester by Environment interaction variance; $\sigma_{\text{ϵ}}^{2}$ , residual variance.

**Table 6:** Summary statistics of GCA estimates of lines and testers for grain yield and other agronomic traits evaluated in multiple locations under drought and optimum conditions.

Statistics	GY		AD		SD		ASI		PH		EH	
	OPT	DS	OPT	DS	OPT	DS	OPT	DS	OPT	DS	OPT	DS
No. of +ve GCA (%)	55	53	51	49	51	44	48	46	52	58	51	52
No. of −ve GCA (%)	45	47	49	51	49	56	52	54	48	42	49	48
Min +ve GCA value	0.004	0.010	0.003	0.02	0.003	0.06	0.001	0.01	0.00	0.04	0.02	0.02
Max +ve GCA value	0.724	1.000	5.059	5.33	4.697	6.18	0.807	1.46	17.83	29.47	12.30	20.29
Min −ve GCA value	−0.99	−0.65	−4.65	−5.42	−4.343	−5.29	−0.687	−0.92	−20.92	−30.25	−15.70	−21.85
Max −ve GCA value	−0.01	−0.01	−0.05	−0.02	−0.023	−0.04	−0.003	−0.01	0	−0.20	−0.09	−0.18
No. of lines	265	245	265	245	265	245	265	245	265	245	265	245
Tester												
No. of +ve GCA (%)	33	50	33	50	33	40	50	50	33	67	50	33
No. of −ve GCA (%)	67	50	67	50	67	60	50	50	67	33	50	67
Min +ve GCA value	0.07	0.002	0.15	0.01	0.15	0.17	0.01	0.03	0.02	0.33	0.29	0.8
Max +ve GCA value	0.07	0.003	0.28	0.40	0.24	0.19	0.03	0.09	2.00	1.06	1.07	1.09
Min −ve GCA value	−0.06	−0.01	−0.15	−0.21	−0.15	−0.23	−0.03	−0.08	−1.83	−1.44	−0.86	−0.96
Max −ve GCA value	−0.01	−0.01	−0.07	−0.06	−0.04	−0.05	−0.01	−0.03	−0.01	−1.34	−0.28	−0.22
No. of testers	6	6	6	6	6	6	6	6	6	6	6	6

GY, grain yield; AD, days to 50% anthesis; SD, days to 50% silking; ASI, anthesis–silking interval; H, plant height; EH, ear height; OPT, optimum; DS, drought stress.

**Figure 3:**
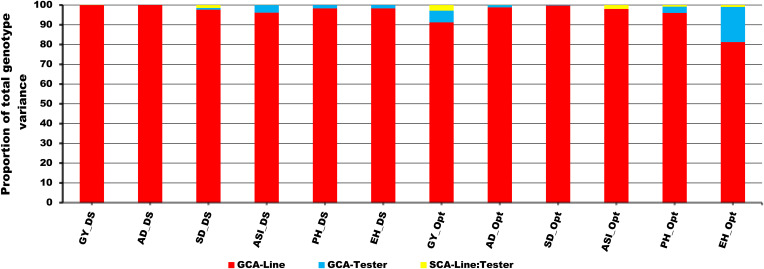
Proportional contribution of GCA-Line, GCA-Tester, and SCA-Line by Tester interaction to total genotype variance of testcrosses for GY and other secondary traits under drought (DS), optimum (Opt), and across all environments (ACR) conditions. The lower bar denoted by red color indicates the GCA-Line; the middle bar denoted by sea blue represents the GCA-Tester, and the upper bar denoted by yellow indicates the SCA-Line by Tester interaction.

The proportional contribution due to GCA variance of lines to the total genotypic variance varied from 81% to 100% under DS and OPT conditions (**Figure 3**). In contrast, the contribution of GCA variance of tester to total genotypic variance ranged from 0% to 4% under DS and from 0% to 18% under OPT conditions (**Figure 3**). Similarly, the SCA variance varied from 0% to 1% under DS compared to 0% to 3% under OPT conditions. Overall, proportional contribution due to GCA variance of lines was by far larger than the contribution due to tester and the interaction between line and tester variance.

### Estimates of GCA and SCA effects under DS and OPT

**Figure 4:**
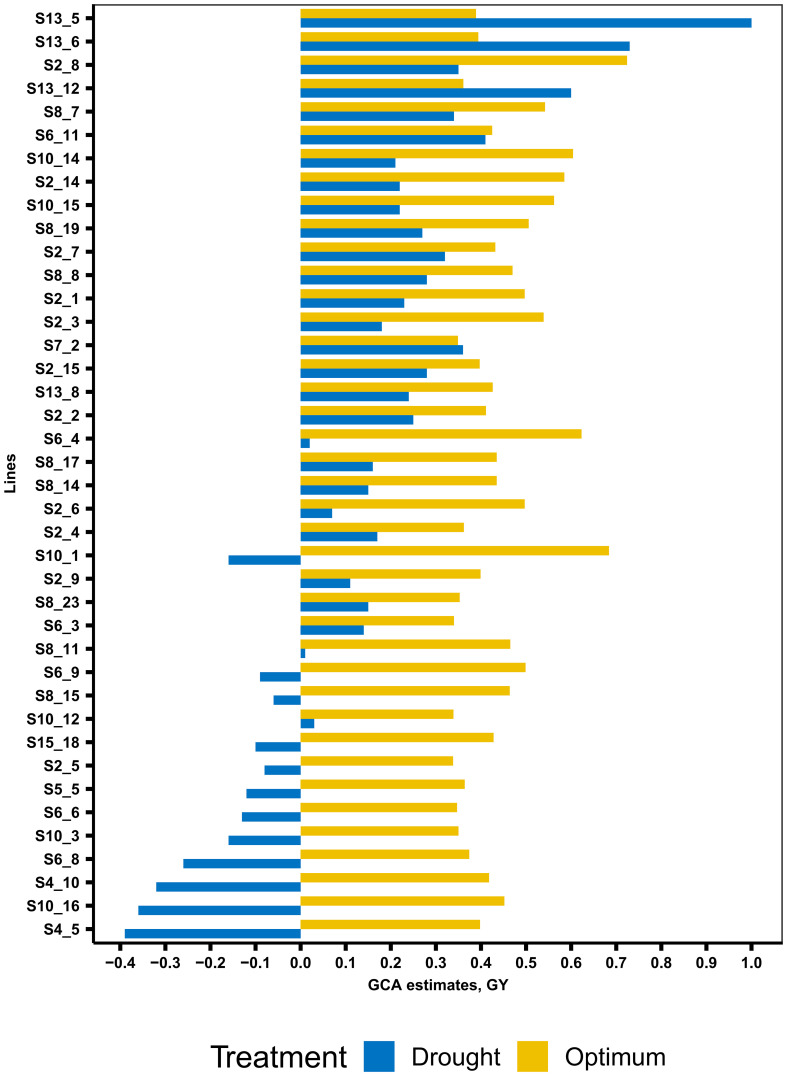
General combining ability estimates of 40 selected best lines for grain yield across managed drought and optimum conditions.

The summary statistics of GCA effects of lines and testers for GY and other traits under DS and OPT conditions is shown in **Figure 4** and in **Tables 6** and **7**. The values of GCA estimates varied among the lines and testers for all the traits. Under DS conditions (**Table 5**), the GCA effect of GY varied from −0.65 to 1.00 with 53% and 47% of lines found to be positive and negative effects, respectively. Lines S13_5, S13_6, S13_12, S6_11, S7_2, S2_8, S8_7, S2_7, S8_8, S2_15, and S8_19 were positive with high GCA effects for GY (**Figure 3**). Tester T5 had the highest GCA effect, whereas tester T4 had the lowest GCA effects for GY. The GCA effects for other traits are also shown in **Tables 6** and **7**.

**Table 7:** Summary statistics of SCA values for grain yield and other agronomic traits evaluated in multiple locations under drought and optimum conditions.

Statistics	GY		AD		SD		ASI		PH		EH	
	OPT	DS	OPT	DS	OPT	DS	OPT	DS	OPT	DS	OPT	DS
No. of positives SCA (%)	49	50	49	49	50	51	51	49	49	52	51	50
No. of negatives SCA (%)	51	50	51	51	50	49	49	51	51	48	49	50
Min positive SCA value	0.001	0.001	0.000	0.001	0.001	0.001	0.000	0.002	0.000	0.026	0.006	0.002
Max positive SCA value	0.455	0.23	1.364	2.657	1.223	3.152	0.547	0.791	6.888	10.741	5.175	7.852
Min negative SCA value	−0.47	−0.18	−1.76	−3.28	−1.10	−2.90	−0.79	−0.76	−5.82	−15.66	−8.43	−11.67
Max negative SCA value	0.000	−0.001	−0.001	−0.001	0.000	−0.001	0.000	−0.001	0.000	−0.013	−0.01	−0.017
No. of testcross	795	735	795	735	795	735	795	735	795	735	795	735

GY, grain yield; AD, days to 50% anthesis; SD, days to 50% silking; ASI, anthesis–silking interval; H, plant height; EH, ear height; OPT, optimum; DS, drought stress.

The GCA effects of ASI ranged from −0.92 (S13_5) to 1.46 (S10_8), while tester GCA effects varied from −0.08 to 0.09 (**Table 7**). More than half of the lines and testers showed a negative GCA effect for ASI. Under OPT conditions (**Figure 3**; **Table 6**), GCA effects varied considerably among the lines and testers. The GCA effects for GY ranged from −0.01 to 0.72. Out of the total lines, 55% of the lines showed positive GCA effects for GY, whereas 45% of the lines displayed negative GCA effects for GY. Line S2_8 recorded the highest GCA effects for GY followed by lines S10_1, S6_4, S10_14, S2_14, S10_15, S8_7, S2_3, S8_19, and S6_9. Testers T1 and T5 showed a positive and high GCA effect for GY. Across DS and OPT management conditions (**Figure 4**; **Tables 6**, **8**), lines S2_8, S10_1, S6_4, S10_14, S2_14, S10_15, S8_7, S2_3, S8_15, and S13_5 consistently exhibited a high and positive GCA effect for GY and a high and negative GCA effect for AD, SD, ASI, PH, and EH.

**Table 8:** GCA estimates of selected best 40 lines and six testers for grain yield and other agronomic traits evaluated in multiple locations under drought and optimum conditions.

Lines	GY		AD		SD		ASI		PH		EH	
	OPT	DS	OPT	DS	OPT	DS	OPT	DS	OPT	DS	OPT	DS
S2_8	0.72*	0.35	−1.05	−0.79	−1.07	−1.28	−0.08	−0.22	11.50**	12.68	6.71*	0.66
S10_1	0.68	−0.16**	0.21	−0.45	−0.19	−0.08	−0.24	−0.1	−0.10	10.37	5.91*	4.09
S6_4	0.62	0.02*	−0.66	2.9**	−0.41	2.91*	0.13	0.11	11.15**	0.04	6.62*	−0.88
S10_14	0.60	0.21	0.95	−0.12	0.59	0.93	−0.16	0.6	−0.07	8.18	5.77*	6.55
S2_14	0.59	0.22	−1.70**	−0.91	−1.40*	−1.51	0.09	−0.32	3.13	4.15	−3.73	−13.02*
S10_15	0.56	0.22	0.824	0.24	0.47	0.82	−0.18	0.4	−0.03	7.75	8.35**	8.12
S8_7	0.54	0.34	−1.66**	0.75	−1.54*	−0.09	−0.02	−0.32	0.001	12.56*	3.32	9.83
S2_3	0.54	0.18	−0.948	−0.08	−1.09	−0.32	−0.15	−0.1	8.70*	2.03	−1.61	−13*
S8_19	0.51	0.27	−2.32**	0.61	−1.72**	−0.06	0.03	−0.22	−0.06	16.98	1.9	8.06
S6_9	0.50	−0.09**	0.56	2.93**	0.14	2.82*	−0.29	0.21	6.83	−10.57	6.328*	−0.84
S2_1	0.50	0.23	−1.46*	−0.31	−1.33*	−0.05	0.01	0.13	11.53**	6.22	4.37	−6.44
S2_6	0.50	0.07	−1.39*	−0.38	−1.55*	−0.69	−0.22	−0.16	5.51	2.04	2.2	−5.93
S8_8	0.47	0.28	−0.87	1.54	−1.21	0.16	−0.23	−0.5	0.04	18.96	7.55**	17.14**
S8_11	0.47	0.01**	−1.35*	0.94	−1.65*	−0.14	−0.29	−0.43	0.00	6.98	4.63	9.32
S8_15	0.46	−0.06**	−2.24**	0.48	−1.59*	1.13	0.31	0.37	−0.01	1.98	−1.02	1.14
S10_16	0.45	−0.36**	1.02	−0.42	0.92	0.95	0.01	0.78	0.07	7.26	12.30**	10.47*
S8_14	0.44	0.15	−2.42**	0.17	−2.46**	−0.53	−0.17	−0.22	−0.03	13.94	1.74	5.56
S8_17	0.44	0.16	−1.54**	0.33	−1.43*	−0.52	−0.02	−0.32	0.02	16.68	5.1	11.24*
S2_7	0.43	0.32	−0.649	0.16	−0.90	−0.09	−0.21	−0.11	7.69*	0.92	1.06	−10.69
S15_18	0.43	−0.10**	3.10**	0.9	2.21**	−0.56	−0.45	−0.69	−0.05	9.15	3.97	10.03*
S13_8	0.43	0.24	1.58**	1.89	1.19	1.3	−0.13	−0.18	−0.04	0.00	3.3	−3.08
S6_11	0.43	0.41	−2.38**	−0.05	−2.49**	0.56	−0.21	0.28	5.03	−11.21	3.90	−6.20
S4_10	0.42	−0.32**	1.1	2.08*	1.15	2.01	0.10	0.01	6.88	−1.17	3.38	5.26
S2_2	0.41	0.25	−1.11	−0.20	−1.09	−0.63	−0.06	−0.16	8.23*	6.46	4.25	−1.63
S2_9	0.40	0.11	−1.77**	−0.09	−1.81**	−0.43	−0.14	−0.16	3.000	−6.97	−0.69	−16.00**
S4_5	0.40	−0.39**	1.20*	3.40**	0.98	2.84*	−0.08	−0.16	1.99	−11.89	3.77	−0.59
S2_15	0.40	0.28	−1.07	−0.57	−0.27	−0.23	0.46	0.13	7.14	8.86	−3.22	−10.19
S13_6	0.39	0.73	−2.10**	−1.6	−2.12**	−3.47**	−0.16	−0.81	−0.08	−7.04	−3.01	−9.16
S13_5	0.39	1.00**	−0.66	−1.63	−1.14	−3.66**	−0.35	−0.92	−0.06	1.06	−0.42	−8.24
S6_8	0.37	−0.26**	−0.89	1.95	−1.28	2.99*	−0.35	0.53	−1.14	−18.9	2.05	−7.61
S5_5	0.36	−0.12**	0.65	0.18	0.45	−0.18	−0.09	−0.19	6.44	9.29	2.93	10.82*
S2_4	0.36	0.17	−1.72**	−0.46	−1.58*	−0.63	−0.01	−0.11	5.89	5.29	−2.38	−11.83*
S13_12	0.36	0.60	−0.59	−0.51	−1.07	−1.68	−0.32	−0.49	−0.04	−2.84	−1.04	−9.19
S8_23	0.35	0.15	−2.03**	−0.35	−2.11**	−0.05	−0.15	0.2	−0.04	−0.37	−2.09	−1.85
S10_3	0.35	−0.16**	0.243	0.39	1.07	1.25	0.09	0.51	−0.03	13.39**	4.84	6.66
S7_2	0.35	0.36	0.003	0.55	0.15	−0.45	0.14	−0.37	0.06	10.02	8.63**	8.38
S6_6	0.35	−0.13**	0.85	2.00	0.23	2.86*	−0.4	0.03	12.19**	−5.49*	9.58**	−0.94
S6_3	0.34	0.14	−1.47*	0.97	−1.65*	0.29	−0.22	−0.29	6.06	−9.56	4.93	−5.13
S10_12	0.34	0.03*	1.111	−0.14	1.36*	1.48	0.25	0.68	0.06	17.00*	8.65**	11.78**
S2_5	0.34	−0.08**	−1.10	−0.09	−1.11	−0.16	−0.08	−0.05	7.74*	−1.02	0.97	−11.49*
SE	0.51	0.32	0.83	0.92	0.94	1.14	0.44	0.56	5.33	7.38	3.81	5.07
Tester												
T1	0.07	0.002	−0.15	−0.21	−0.09	−0.05	0.03	0.05	2.00**	1.06	0.57	−0.42
T2	−0.06	−0.002	−0.12	0.01	−0.15	0.19	−0.02	0.09	−0.18	−1.44	0.29	1.09
T3	−0.01	−0.001	0.28**	−0.06	0.24*	0.00	−0.01	0.03	−1.83**	−1.34	−0.86*	−0.96
T4	−0.03	−0.004	0.15	−0.19	0.15	−0.08	0.01	−0.03	0.02	0.51	1.07*	−0.3
T5	0.07	0.003	−0.08	0.05	−0.04	−0.23	0.02	−0.07	−0.01	0.88	−0.78	0.8
T6	−0.05	0.002	−0.07	0.40	−0.11	0.17	−0.03	−0.08	−0.01	0.33	−0.28	−0.22
SE	0.08	0.001	0.13	0.002	0.14	0.18	0.01	0.11	0.80	1.36	0.58	0.90

*, **, Significant at *p* < 0.05 and < 0.01 probability levels, respectively. OPT, optimum; DS, drought stress; GY, grain yield; AD, days to 50% anthesis; SD, days to 50% silking; ASI, anthesis–silking interval; H, plant height; EH, ear height.

**Figure 5:**
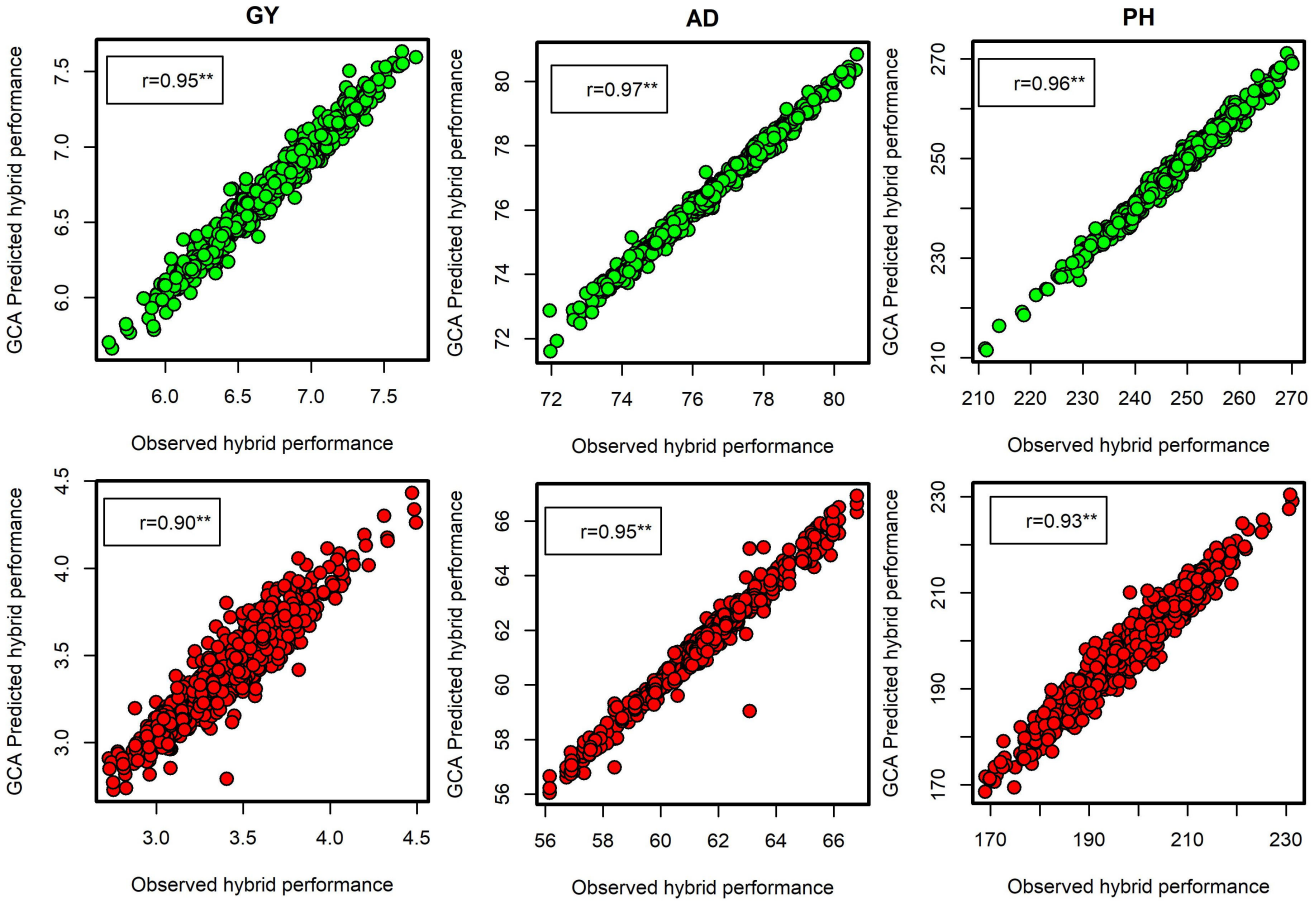
Leave-one-hybrid-out cross-validated *r* values between general combining ability (GCA)-based predicted testcross hybrid performance and observed hybrid performance for grain yield, anthesis date, and plant height evaluated under optimum (green dots) and managed drought conditions (red dots).

The summary statistics of SCA effects on selected testcross hybrids for all the traits evaluated under DS and OPT conditions is presented in **Tables 7** and **9**. Across all the management conditions, some of the selected testcross hybrids showed consistently high and positive SCA effects for GY and a high negative SCA effect for other traits. This indicates that these testcross hybrids are good specific combiners for GY and possess desirable plant architectural characteristics including shorter number of days to anthesis and silking, reduced period between anthesis and silking, shorter plant stature, and lower ear placement. Prediction correlation between the observed testcross hybrid performance and the GCA-predicted hybrid performance was high for GY with *r* = 0.95 under OPT and *r* = 0.90 under DS conditions (**Figure 5**). Prediction correlations were also high for AD and PH under both OPT and managed DS conditions.

**Table 9:** SCA estimates of selected best 40 lines-by-tester combinations for grain yield and other agronomic traits evaluated in multiple locations under drought and optimum conditions.

Testcross	GY		AD		SD		ASI		PH		EH	
	OPT	DS	OPT	DS	OPT	DS	OPT	DS	OPT	DS	OPT	DS
S6_7 × T4	0.455	−0.008	−0.417	−0.488	−0.291	0.569	0.083	0.394	−0.056	4.364	−1.628	−1.596
S15_7 × T2	0.44	0.025	−0.424	0.253	−0.381	0.386	0.029	0.141	0.026	−1.442	3.372	0.211
S3_7 × T6	0.376	0.002	−0.446	0.384	−0.231	0.553	0.135	0.076	1.57	1.218	−1.623	−1.161
S8_6 × T2	0.3	0.011	−0.195	−0.492	−0.388	−0.243	−0.133	0.064	−0.019	4.197	2.083	4.034
S3_5 × T6	0.296	−0.038	0.188	−0.831	0.287	−0.334	0.073	0.148	−0.423	6.728	−0.612	3.358
S15_6 × T2	0.288	0.023	0.253	−0.059	0.328	0.964	0.054	−0.038	−0.024	7.514	1.596	7.465
S1_16 × T6	0.284	0.00	−0.173	0.159	−0.189	−0.001	−0.034	−0.019	−1.032	−0.291	−0.221	−1.326
S5_9 × T6	0.272	0.022	−0.045	0.261	0.159	−0.275	0.115	−0.263	−1.238	−2.851	−1.06	−0.78
S3_13 × T6	0.262	0.027	−0.643	−0.715	−0.548	−0.722	0.038	−0.053	2.796	1.211	1.768	1.595
S4_10 × T1	0.258	−0.046	−0.25	0.408	−0.112	0.167	0.088	−0.095	2.766	−4.17	2.119	−0.706
S4_5 × T4	0.236	−0.003	−0.026	−0.594	−0.066	−0.208	−0.035	0.133	−0.637	2.708	0.148	1.014
S2_10 × T6	0.236	−0.017	−0.081	0.177	−0.279	−0.055	−0.135	−0.111	0.536	0.589	−0.483	1.343
S1_17 × T1	0.235	0.034	0.373	0.086	0.22	0.17	−0.079	0.009	2.667	2.38	−0.548	0.848
S2_13 × T4	0.234	0.011	0.193	−3.277	0.18	−2.904	−0.031	−0.121	2.086	2.715	0.65	2.983
S5_13 × T6	0.228	−0.003	0.211	0.584	0.31	−0.024	0.062	−0.294	−2.228	0.66	0.008	2.917
S15_14 × T2	0.209	0.015	0.356	0.345	0.848	0.152	0.366	−0.061	0.017	0.303	1.332	−1.493
S7_9 × T2	0.061	0.158	−0.037	−0.503	−0.12	−1.48	−0.068	−0.435	−0.01	10.741	−0.858	1.462
S12_4 × T2	0.06	0.03	−0.049	0.656	0.157	0.622	0.135	−0.002	0.021	9.629	−0.765	4.599
S14_4 × T2	0.059	0.066	−0.335	−0.503	−0.503	−0.64	−0.146	−0.111	0.012	−4.776	−1.084	−8.705
S11_7 × T3	0.058	0.039	0.261	−0.139	0.468	−0.806	0.158	−0.283	0.008	−0.621	−1.413	−2.034
S4_3 × T4	0.05	0.018	−0.085	−0.137	−0.418	−0.655	−0.215	−0.235	−1.506	−0.019	−0.471	2.541
S5_4 × T4	0.05	0.064	0.175	0.368	−0.029	−0.265	−0.149	−0.275	1.988	−0.013	3.661	−1.215
S1_13 × T6	0.049	0.008	−0.369	0.139	−0.359	−0.404	−0.04	−0.192	−0.011	−5.232	−0.316	−3.455
S12_10 × T2	0.049	0.063	−0.209	−0.141	−0.461	−0.024	−0.155	−0.19	−0.048	2.073	−1.34	−2.576
S6_11 × T4	0.044	0.071	−0.286	−0.658	−0.165	−0.866	0.059	−0.158	0.125	6.122	−1.22	2.958
S3_14 × T6	0.043	0.037	−0.303	−0.513	−0.427	−0.479	−0.126	−0.007	4.376	−0.503	1.077	0.196
S8_13 × T2	0.042	0.029	−0.627	0.104	−0.398	−0.509	0.103	−0.228	−0.009	0.599	−0.611	−0.12
S6_6 × T1	0.041	0.039	−0.04	1.24	−0.125	−0.128	−0.044	−0.244	−0.13	6.539	−0.434	7.098
S4_11 × T4	0.037	0.002	0.534	0.037	0.192	−0.424	−0.194	−0.189	2.037	1.619	3.027	3.611
S7_8 × T2	0.036	0.077	−0.047	−0.617	−0.09	−1.761	−0.071	−0.477	−0.031	1.067	−0.213	−2.509
S1_11 × T1	0.029	0.035	−0.068	0.144	0.149	0.281	0.195	0.059	2.703	−0.587	2.425	−2.27
S12_2 × T5	0.026	0.005	0.201	0.025	0.469	−0.032	0.185	0.165	−0.019	−4.107	1.399	−2.522
S11_9 × T2	0.02	0.027	0.248	−1.42	0.302	−1.925	0.05	−0.344	0.076	2.019	−0.437	1.844
S13_1 × T2	0.016	0.011	0.639	0.411	0.653	0.27	0.067	−0.009	0.034	−0.926	−0.862	0.464
S2_15 × T1	0.014	0.015	−0.064	0.2	0.269	−0.175	0.198	−0.156	0.491	−2.025	0.454	−1.04
S4_2 × T6	0.012	0.034	0.034	−0.137	0.095	−0.541	0.009	−0.149	−0.589	−0.986	0.635	−4.07
S2_16 × T4	0.011	0.073	0.195	0.418	0.115	−0.201	−0.05	−0.197	−0.882	−0.881	0.483	−1.605
S2_16 × T6	0.009	0.05	−0.088	−0.437	−0.011	−0.495	0.047	−0.043	0.319	4.526	−0.776	4.714
S5_14 × T1	0.006	0.033	−0.419	−0.947	−0.134	−0.845	0.206	−0.012	0.75	−4.241	−0.771	−3.113
S6_1 × T1	0.006	0.061	0.787	0.299	0.73	−0.246	0.015	−0.233	0.398	7.972	1.507	3.695

GY, grain yield; AD, days to 50% anthesis; SD, days to 50% silking; ASI, anthesis–silking interval; H, plant height; EH, ear height; OPT, optimum condition; DS, drought condition.

## Discussion

Drought stress is one of the main factors for yield losses in maize production. Yield improvement under DS management has become a “must have trait” in tropical maize breeding programs in SSA. In this study, the significant genotype variability among the testcross hybrids for GY and other agronomic traits under DS and optimum conditions indicated that trait improvements could be achieved through selection. Therefore, these parental lines could further be exploited for yield improvement under OPT and DS conditions (Ertiro et al., 2017; Santos et al., 2019).

The present study showed that environment and GEI variance components were significant for GY and other agronomic traits, suggesting that hybrids responded differentially across both DS and OPT environments. These results agree with those previous findings by Sserumaga et al. (2016, 2021) and Sorsa et al. (2023). The significant GEI effects on the expression of a trait can obscure the correlation between genotype and phenotype values, leading to reduced selection response (Yan, 2014). This implies that the ability to identify high-yielding and consistently performing hybrids across DS and OPT growing environments will be hampered by the high GEI variance. Therefore, rigorous testing of hybrids across multiple locations and over several years is essential before hybrids can be recommended for cultivar release. This result is in agreement with the findings of several studies (Sserumaga et al., 2016; Annor et al., 2019; Sorsa et al., 2023; Ndlovu et al., 2024, 2021).

High genotypic variance and moderate to high broad-sense heritability estimates were observed for AD, SD, ASI, EH, and PH under drought and optimum. However, heritability for GY was low under drought. The low heritability of GY observed under the drought experiment suggests that secondary traits exhibiting high heritability may enhance the effectiveness of selection responses (Lu et al., 2011; Rezende et al., 2020). These results are in agreement with previous studies that reported lower genotypic variances and heritabilities for GY under stressed conditions (Badu-Apraku and Fakorede, 2017; Banziger et al., 1997; Beyene et al., 2013).

The increased mean value of ASI observed under DS, the significant yield reduction and increased AD and SD, and decreased PH relative to the OPT condition implied that the intensity of DS was sufficient enough to discriminate among testcross hybrids for drought tolerance (Sang et al., 2022). Previous studies have demonstrated that a significant decrease in GY, ranging from 20% to 30%, is a clear indication of severe DS (Edmeades et al., 1999, 2015; Sang et al., 2022). The observed decrease in GY in the current study is in agreement with previous studies reported by Trachsel et al. (2016); Ertiro et al. (2017); Annor et al. (2020), and Matongera et al. (2023). A study conducted by Ertiro et al. (2017) on testcross performance for their combining ability under DS and low nitrogen reported a 50% yield reduction and an increase in ASI by 149% with a 1% decrease in PH. The reduction of GY under DS can be attributed to increased ASI leading to high yield reduction among the testcross hybrids. DS inhibits silk development, thus delaying the silking process, leading to increased period between anthesis and silking resulting in higher rates of ear and kernel abortion, which can cause ear barrenness, leading to GY reduction (Bänziger et al., 2000).

Direct selection of testcross hybrids for GY alone under DS is considered inefficient due to the observed low heritability and low genetic variance (Ertiro et al., 2017; Kim et al., 2017). On the other hand, ASI can be used as a secondary trait for indirect selection of genotypes with improved drought tolerance and high GY in maize breeding (Bänziger et al., 2000; Betrán et al., 2003a; Cooper et al., 2014). The mean performance of GY and other traits in the 30 selected testcross hybrids was generally higher than the mean performance of the best commercial checks across DS and OPT conditions. This suggested that the testcross possesses high mean performance for drought tolerance. Similar observations were reported in previous studies (Sserumaga et al., 2016; Ertiro et al., 2017; Matongera et al., 2023).

Understanding the trait correlations across DS and OPT conditions can provide valuable insights for making informed decisions in breeding for drought tolerance (Badu-Apraku et al., 2012; Ziyomo and Bernardo, 2013). It is possible to improve positively correlated traits by focusing on the improvement of one easily measurable trait. According to Bänziger et al. (2000), simultaneous selection of drought tolerance and GY under DS can be efficiently implemented by prioritizing secondary traits that are both easily measurable and highly correlated with GY under managed drought conditions. The presence of significant negative association between GY and flowering traits (AD, SD, and ASI) under DS and OPT conditions suggested that these traits have indirect effects on GY across the management conditions. These results are in agreement with the findings of previous studies (Badu-Apraku et al., 2012; Ertiro et al., 2017, 2022; Talabi et al., 2017; Oyekunle and Badu-Apraku, 2018; Matongera et al., 2023).

The prevalence of GCA (line and tester) variance over SCA for GY and other agronomic traits within and across management conditions implied that additive gene action for the inheritance of these traits was higher than nonadditive gene effects. These findings corroborate previous results reported by Betrán et al. (2003b), Makumbi et al. (2011); Amegbor et al. (2017); Ertiro et al. (2017), and Oluwaseun et al. (2022). The current study results also contradict those of de Souza et al. (2009) and Annor et al. (2019) who reported that nonadditive effects predominantly governed GY and other secondary traits under DS conditions. The difference in the outcomes of the current study compared to previous studies can likely be attributed to the differences in the germplasms utilized.

In the present study, the Baker’s variance ratio showed that there is a high probability of predicting testcross hybrid performance based on GCA variance (line or tester) alone under DS and OPT conditions. The ratios closer to or equal to one implied that the traits are governed predominantly by additive gene action and that there is a high likelihood of predicting testcross hybrid performance based on only GCA effects. This is well supported by the observed high correlations between testcross hybrid performance and GCA-predicted hybrid performance for GY, AD, and PH under both OPT and DS conditions (**Figure 5**). This further suggested that additive effects had a more significant impact on all the traits than non-additive effects. The results of this study corroborates the findings of Amegbor et al. (2017) and Oluwaseun et al. (2022).

The estimates of combining ability effects hold great significance as they reveal the inherent ability of a set of genotypes to effectively pass on desirable genes to their offspring. This valuable information aids in making informed decisions regarding genetic selection and enhancement (Hallauer et al., 2010). According to several researchers (Makumbi et al., 2011; Ertiro et al., 2017; Annor et al., 2019), inbred lines possessing favorable GCA effects for GY and secondary traits are highly valuable in breeding programs. These lines can be utilized (i) as parental lines and incorporated into recurrent selection programs to enhance the overall genetic potential of future generation, (ii) as excellent parent candidates for developing synthetics if necessary, and (iii) for recycling to maintain their superior genetic attributes within breeding populations. Generally, inbred lines with a high GCA can be utilized as excellent broad-based testers. In this regard, inbred lines S2_8, S10_1, S6_4, S10_14, S2_14, S10_15, S8_7, S2_3, S8_15, and S13_5 consistently exhibited high and desirable GCA effects for GY and other agronomic traits (**Table 8**). Similarly, tester 5 exhibited high GCA effects for GY and other secondary traits. Lines with negative GCA effects for AD, SD, and ASI are suitable for exploiting desirable genes for developing early maturity cultivars with short period between male and female flowering, a key measure of drought tolerance. High negative GCA effects for PH and EH suggest that these lines possess shorter plant and ear height genes, which could be selected as parental lines for exploiting lodging resistance. The high and desirable GCA effects shown by inbred lines suggested that they possess necessary sources of genes/alleles for high GY, earliness, shorter ASI, reduced PH, and lower ear placement that could potentially be transmitted to offspring through breeding.

The following testcross combinations consistently showed a high positive SCA effect for GY and a high negative SCA effect for other traits across both DS and OPT conditions: S7_9 × T2, S7_8 × T2, S2_16 × T4, S6_11 × T4, S12_10 × T2, S6_1 × T1, S3_13 × T6, S15_7 × T2, S15_6 × T2, S5_9 × T6, S4_3 × T4, S8_6 × T2, S1_13 × T6, and S3_7 × T6. These findings suggested that the testcrosses are excellent candidates for improving GY with desirable agronomic traits under both drought and optimum conditions. The observed high correlation between testcross hybrid performance and GCA-predicted hybrid performance for GY, AD, and PH under both optimum and DS conditions indicates the predominance of GCA effects (**Figure 5**). Furthermore, this result gives an opportunity for breeders to test a greater number of lines without increasing the number of plots and resources even for both optimum and DS conditions.

## Conclusion

A line × tester experiment of 765 testcross hybrids and six commercial checks was evaluated under managed OPT and DS conditions. In the present study, we observed significant genetic variations among the testcross hybrids for GY and other agronomic traits under both OPT and DS conditions, which supports the possibility of genetic improvement through selection. Combining ability estimates and Baker’s ratio indicate a preponderance of additive gene action over non-additive gene action. Therefore, significant progress can be achieved through recurrent selection. Ten inbred lines (S2_8, S10_1, S6_4, S10_14, S2_14, S10_15, S8_7, S2_3, S8_15, and S13_5) consistently showed a high and desirable GCA effect for GY and other traits across DS and OPT conditions. These lines could be used as donors for the improvement of drought tolerance or for introgression of their favorable alleles into other elite lines through recurrent selection. Fourteen testcross hybrids (S7_9 × T2, S7_8 × T2, S2_16 × T4, S6_11 × T4, S12_10 × T2, S6_1 × T1, S3_13 × T6, S15_7 × T2, S15_6 × T2, S5_9 × T6, S4_3 × T4, S8_6 × T2, S1_13 × T6, and S3_7 × T6) were identified with high yield potential and with other desirable agronomic traits under DS and OPT conditions. These testcross hybrids should be further evaluated extensively under on-farm conditions, which mimic the real smallholder farmers’ conditions and confirm their consistent performance before releasing for commercialization.

## Data Availability

The datasets presented in this study can be found in online repositories. The names of the repository/repositories and accession number(s) can be found in the article/**Supplementary Material**.
