# Corrigendum: Testcross performance and combining ability of intermediate maturing drought tolerant maize inbred lines in Sub-Saharan Africa

**DOI:** 10.3389/fpls.2025.1627898

**Published:** 2025-08-14

**Authors:** Kulai Amadu Manigben, Yoseph Beyene, Vijay Chaikam, Pangirayi B. Tongoona, Eric Y. Danquah, Beatrice E. Ifie, Isaiah Aleri, Andrew Chavangi, Boddupalli M. Prasanna, Manje Gowda

**Affiliations:** ^1^ International Maize and Wheat Improvement Center (CIMMYT), World Agroforestry Centre (ICRAF), United Nations Avenue, Nairobi, Kenya; ^2^ West Africa Centre for Crop Improvement (WACCI), University of Ghana, Accra, Ghana; ^3^ Maize Improvement Program (MIP), The Council for Scientific and Industrial Research (CSIR)-Savanna Agricultural Research Institute, Tamale, Ghana

**Keywords:** drought, grain yield, general combining ability, line-by-tester design, specific combining ability, maize

In the published article, there was an error in **Figure 1** and **3** as published. **Figures 1** and **3** were interchanged.

The corrected **Figure 1** and its caption are shown below:

**Figure 1:**
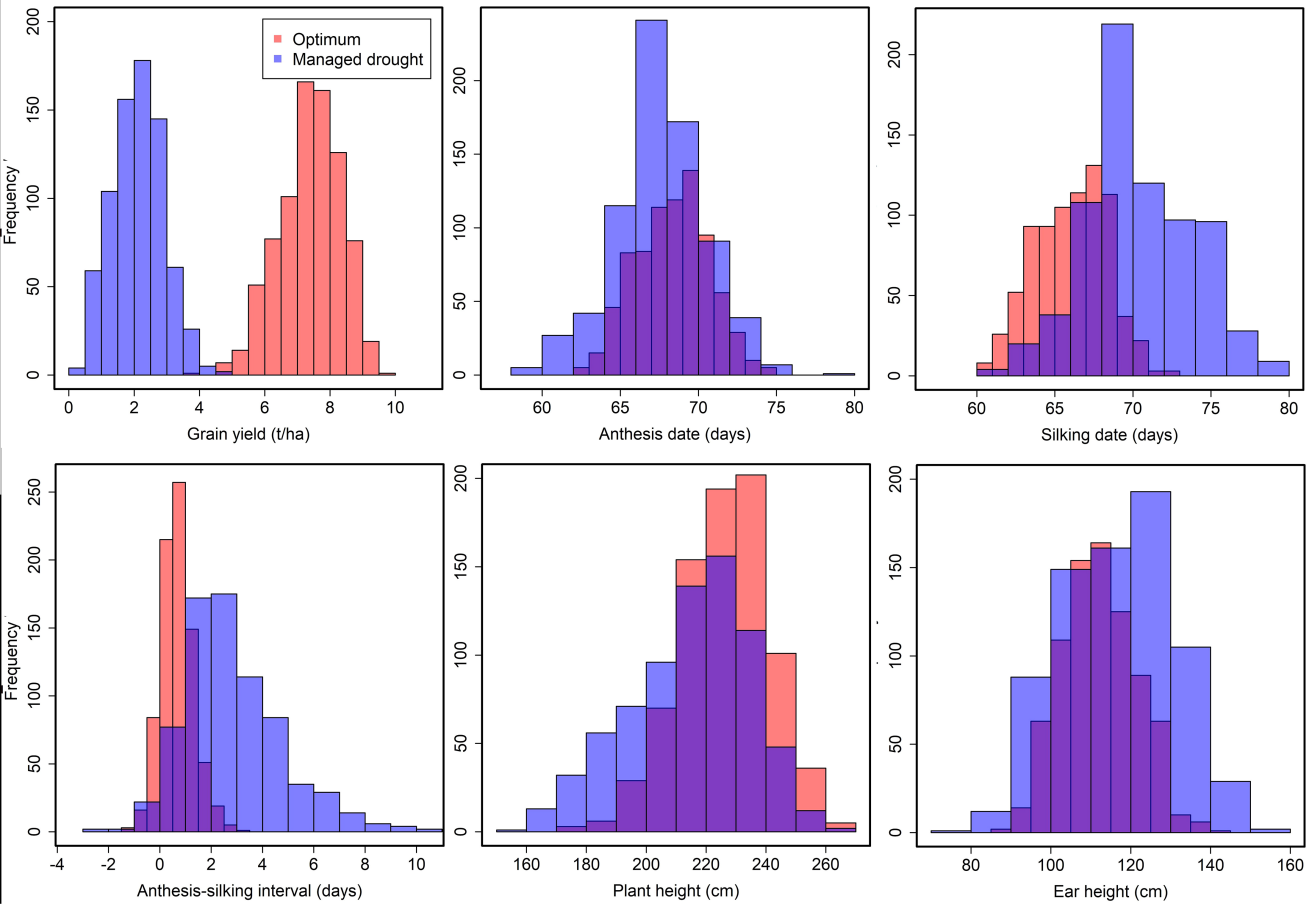
Frequency distribution of grain yield, anthesis date, silking date, anthesis–silking interval, plant height, and ear height recorded under managed drought and optimum conditions. Blue color denotes traits measured under drought stress and orange color denotes trait performance under optimum condition.

The corrected **Figure 3** and its caption are shown below:

**Figure 3:**
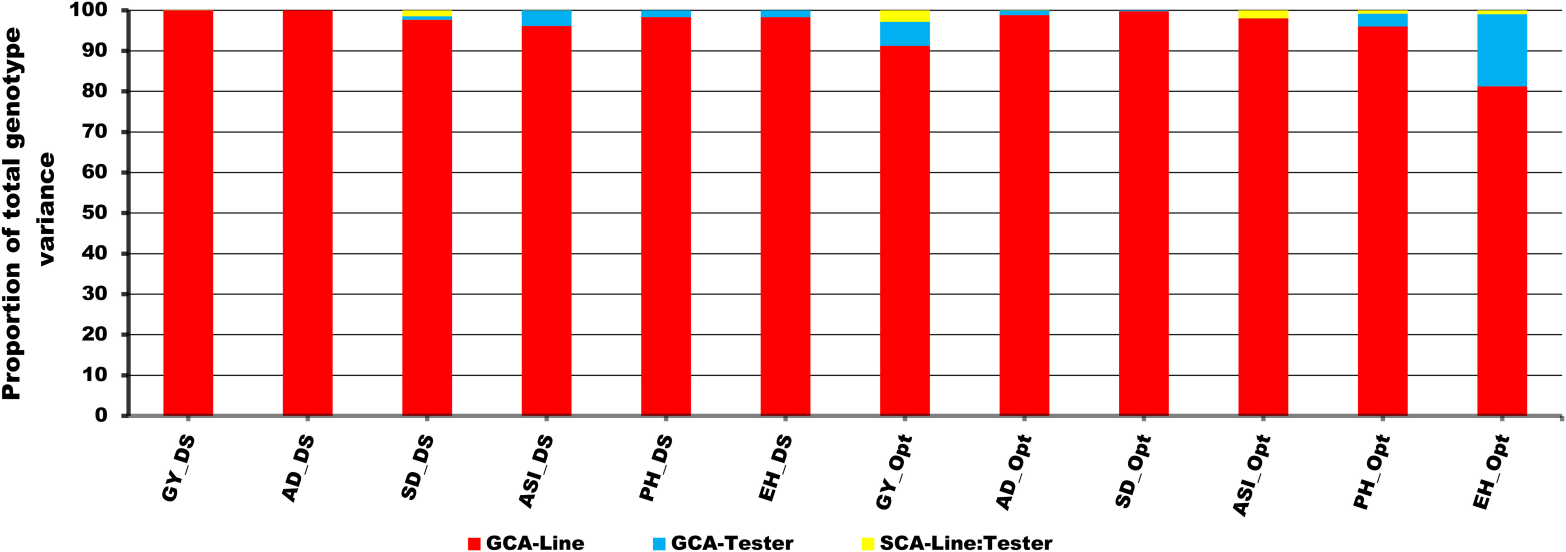
Proportional contribution of GCA-Line, GCA-Tester, and SCA-Line by Tester interaction to total genotype variance of testcrosses for GY and other secondary traits under drought (DS), optimum (Opt), and across all environments (ACR) conditions. The lower bar denoted by red color indicates the GCA-Line; the middle bar denoted by sea blue represents the GCA-Tester, and the upper bar denoted by yellow indicates the SCA-Line by Tester interaction.

The authors apologize for this error and state that this does not change the scientific conclusions of the article in any way. The original article has been updated.

